# NME2 Reduces Proliferation, Migration and Invasion of Gastric Cancer Cells to Limit Metastasis

**DOI:** 10.1371/journal.pone.0115968

**Published:** 2015-02-20

**Authors:** Yan-fei Liu, Aijun Yang, Wei Liu, Chenyu Wang, Min Wang, Lihan Zhang, Dongcang Wang, Jing-fei Dong, Min Li

**Affiliations:** 1 Institute of Pathology, School of Basic Medical Sciences, Lanzhou University, Lanzhou, China; 2 Department of Pathology, Xi’an Children’s Hospital, Xi’an, China; 3 Puget Sound Blood Center, Seattle, Washington, United States of America; 4 Division of Hematology, Department of Medicine, University of Washington, School of Medicine, Seattle, Washington, United States of America; 5 Key Laboratory of Preclinical Study for New Drugs of Gansu Province, Lanzhou University, Lanzhou, China; Stony Brook University, UNITED STATES

## Abstract

Gastric cancer is one of the most common malignancies and has a high rate of metastasis. We hypothesize that *NME2* (Nucleoside Diphosphate Kinase 2), which has previously been considered as an anti-metastatic gene, plays a role in the invasiveness of gastric cancer cells. Using a tissue chip technology and immunohistochemistry, we demonstrated that NME2 expression was associated with levels of differentiation of gastric cancer cells and their metastasis into the lymph nodes. When the *NME2* gene product was over-expressed by ;*in vitro* stable transfection, cells from BGC823 and MKN45 gastric cancer cell lines had reduced rates of proliferation, migration, and invasion through the collagen matrix, suggesting an inhibitory activity of NME2 in the propagation and invasion of gastric cancer. NME2 could, therefore, severe as a risk marker for gastric cancer invasiveness and a potential new target for gene therapy to enhance or induce NME2 expression.

## Introduction

Cancer remains as a leading cause of death, accounting for 14.1 million new cases and 8.2 million deaths in 2012 [1]. The numbers of new cases are expected to increase 70% worldwide to 22 million within the next two decades [2]. Cancers in the lungs, stomach, liver, colon and breasts have the highest mortality [3]. Gastric cancer cells can directly spread to adjacent organs (local invasion) such as the pancreas, the transverse colon, the liver and the spleen as well as to remote lymph nodes, the lungs, and bone tissue. While being two different pathological processes, local invasion and remote metastasis are interconnected where the former often promotes and propagates the latter. Genetic mutations and their aberrant products are key hallmarks and enablers of cancer cells for proliferation, resistance to apoptosis, local invasion, metastasis, immune evasion, angiogenesis, and response to DNA damage. *NME* (Nucleoside Diphosphate Kinase 2 or Non-Metastatic Cells) represents a group of cancer-associated and/or cancer-regulating genes.

*NME* consists of a family of 10 genes that are also known as the *NM23* genes [4] and has been associated with suppressing cancer metastasis and invasion to local tissue [5]. Among the members of this gene family, *NME1* and *NME2* have been extensively studied for their cancer-suppressing activities. *NME2*, which is mapped on chromosome 17q21 [6,7], encodes the B subunit of the nucleoside diphosphate (NDP) kinase [4]. Its product has been reported to inhibit the metastasis of breast cancer and melanoma cells; and a decrease in its expression was correlated with a greater metastatic potential of these cancers [8,9]. However, *NME2* overexpression was also associated with poor prognosis for neuroblastoma and osteosarcoma [10,11]. Moreover, the *NME2* gene was not correlated with the metastasis of endometrial hepatocellular and thyroid carcinoma [12–14]. These apparently conflicting data suggest that NME2 may have differential effects on different types of cancer cells and their ability to locally invade surrounding tissues or metastasize to remote organs.

Because of these diverse NME2 activities on different cancer types, we analyzed tissues surgically removed from patients with gastric cancer and associated the NME2 expression in these tissues with their pathological characteristics. This pathology study was complemented by *in vitro* experiments, where we examined rates of proliferation, migration and invasion of gastric cancer cells that have been stably transfected with a human *NME2* cDNA to overexpress its product. These *in vitro* experiments are designed to understand the invasiveness of cancer cells that express different levels of NME2.

## Materials and Methods

This study was approved by the ethic committee on conducting human research of Lanzhou University, School of Medicine. Patients or their guardians provided their written informed consent before being recruited into the study.

### Tissue immunohistochemistry

We analyzed NME2 expression in gastric cancer and adjacent normal tissues surgically removed from 139 patients admitted to the Army Regional Hospital in the city of Lanzhou from 2011 to 2012. No patients received chemotherapy or radiation therapy before surgery. These surgically removed cancer tissues were processed for hematoxylin and eosin (HE) staining. Briefly, a wax module was punched 42 aperture holes (6×7 holes/chip) of 2 mm each. Tissue blocks were embedded in these holes (one sample/hole). This wax module was then sectioned so that tissues from 40 patients could be examined simultaneously on a single slide to ensure stain uniformity. For control, first and last holes were embedded with tissue from the non-cancerous liver and kidney, respectively. NME2 expression was detected using a commercial immunohistochemistry kit (Beijing ZSGB Biotechnology Co, Ltd.) with a rabbit anti-human NME2 antibody (1:300 dilution, Beijing Biosynthesis Biotechnology Co, Ltd.) according to the manufacturer’s instructions. Levels of NME2 expression were numerically scored as shown in Table 1 by a pathologist who was blinded to the clinical information of these patients.

**Table 1:** A correlation between *NME2* expression and histology grads of gastric carcinomas.

Pathological parameters	NME2expression	r	P
Differentiation		0.436	0.000**
Well differentiated(n = 5)	2.90±0.22		
Moderately differentiated(n = 68)	2.32±0.46		
Poorly differentiated and mucinous adenocarcinoma (n = 66)	1.97±0.45		
Lymph node metastasis		-0.281	0.001**
N_0_ (n = 33)	2.35±0.42		
N_1_ (n = 56)	2.23±0.55		
N_2_ (n = 37)	2.02±0.43		
N_3_ (n = 13)	1.93±0.50		

The number of lymph node metastasis: N_0_ = 0, 0*<*N_1_ *<7*, *7*≤N_2_ *<16*, N_3_≥16. Data were analyzed by a Pearson correlation analysis, **P<0.01.

### Cell culture and cDNA transfection

Cells from the BGC823 and MKN45 gastric cancer cell lines were purchased from the Chinese Academy of Science (Shanghai, China). Cells in the BGC823 line were originally from a 62-year-old man with poorly differentiated adenocarcinoma of the stomach and those in the MKN45 line derived from a metastatic mass of the liver from a 62-year-old Japanese woman with an undifferentiated adenocarcinoma of the stomach. Cells from both lines had residual levels of NME2 expression and are, therefore, ideal for studying the impact of overexpressing this gene product on gastric cancer cells *in vitro*.

The cells were cultured in a DMEM medium (Sigma, St. Louis, MO, US) containing 10% of fetal calf serum (Hangzhou Sijiqing Biological Engineering Materials Co., Ltd., Hangzhou, China) at 37°C with 5% CO_2_ and 95% air. At 70–80% of confluence, these cells were transfected with a cDNA for the human *NME2* gene that was cloned into a pcDNA3.1 vector (Shanghai GenePharma Co.,Ltd. Shanghai, China) using lipofectamine 2000 as the DNA carrier (Invitrogen, Grand Island, NY, US). Cells stably expressing NME2 (designated as NME2) were selected by growing the transfected cells in the DMEM medium containing 1 mg/ml of G418. Control cells were transfected with the pcDNA vector without the *NME2* cDNA insert (designated as mock) and underwent the same selection. Un-transfected parental cells were also examined as baseline controls. An overexpression of NME2 was determined by RT-PCR to quantify the level of *NME2* mRNA and by immunoblots of transfected cell lysates using a polyclonal NME2 antibody (1:100 dilution, Beijing Biosynthesis Biotechnology Co, Ltd.)

### RNA isolation and RT–PCR

Total RNA was isolated from transfected and non-transfected cells using a commercial RNA isolation kit (TaKaRa Biotechnology, Dalian, Liaoning, China) according to the manufacturer’s instructions. RNA (2 μg) from each sample was reverse-transcribed using a PrimeScript RT Kit following the manufacturer’s protocol. The complementary DNA reversely transcribed from the RNA extracted from these cells was amplified by a Taq polymerase using the following primers for the *NME2* cDNA: 5’-AAGCAGCACTACATTGACCTGAAA-3’ (forward) and 5’-GGTCTCCCCAAGCATCACTC-3’ (reverse). Glyceraldehyde 3-phosphate dehydrogenase (GAPDH) was also amplified as control using the following primers: 5’-GCACCGTCAAGGCTGAGAAC-3’ (forward) and 5’-TGGTGAAGACGCCAGTGGA-3’ (reverse). The amplification reaction was initiated by denaturing DNA at 95°C for 5 min, followed by 30 cycles of template denaturing at 94°C for 1min, primer annealing at 60°C for 1min, and primer extension at 72°C for 1min. The NME2 overexpression was quantitatively defined as an increase in *NME2* mRNA by two fold or greater from the baseline set by sham transfected cells.

### Immunofluorescence staining of the *NME2* product

Cells stably transfected with the *NME2* cDNA and a vehicle vector were plated on glass coverslips and allowed to grow until confluence. They were rinsed twice with ice-cold phosphate-buffered saline (PBS), fixed in 4% of paraformaldehyde in PBS for 15min, permeabilized with 0.5% of Triton X-100, and incubated with 1% of bovine serum albumin (BSA) for 30 min to block non-specific binding. The cells were then incubated with a rabbit antibody against human NME2 (1:100, Beijing Biosynthesis Biotechnology) for 24 hr at 4°C followed by incubation with a FITC-conjugated secondary antibody (1:100, Beijing Biosynthesis Biotechnology) for 1 hr. All antibodies were diluted in PBS containing 1% BSA. Nuclei were counterstained with DAPI (1:100 in PBS, 10 min at room temperature).

### Cell cycle analysis

Cells stably expressing NME2 and control cells in culture were collected and fixed with 70% of ice-cold ethanol at 4°C for 24 hr, washed twice with ice-cold PBS, and then treated with RNase A (20 μg/ml) for 30 min at 37°C. They were incubated with propidium iodide (10 μg/ml of final concentration) in the dark for 30 min at room temperature before analysis by flow cytometry for DNA ploidy.

### Colony-formation assay

Transfected and control cells were cultured at an initial density of 1,000 cells per 100-mm culture dish in a complete DMEM medium for up to 4 weeks. They were then stained with methyl violet. All cell colonies that were larger than 2 mm in diameter (contained ≥50 cells) were counted in three separate dishes and expressed as mean ± SEM. For the purpose of data validation, we also counted viable cells that had been cultured for up to 96 hrs. Briefly, parental BCG823 and MKN45 cells and cells that were stably transfected with either a human *NME2* cDNA or the vehicle vector (pc-DNA 3.1) were plated at a density of 2 x 10^4^ cells/ml. They were detached 48–96 hrs after initial seeding with 1% trypsin and centrifuged at 1600 x g. Cell pellets were re-suspended in PBS and incubated with 0.04% of trypan blue for 3 min. Surviving cells were counted on a hemocytometer.

### Assays for cell migration

Two complementary assays were performed to measure the impact of overexpressing NME2 on the rate of cell migration. First was a wound-healing assay, where cells were plated in 6 well plates and allowed to grow to ≥ 95% confluent. After washing cells twice with PBS, a sterile pipette tip was used to scratch the cell monolayer (4–5 parallel scratches/plate). Cells were washed again with PBS, photographed to mark scratched tracks, and incubated with 2.5 ml of serum-free DMEM medium. At a baseline rate of cell migration, a scratched area was partially covered by cells migrated into the injured area 24–48 hr after injury, resulting in a smaller cell-free area. Because this wound-healing assay is semi-quantitative, the results were further validated by a Transwell cell migration assay. Briefly, 6×10^5^ cells suspended in 500 μl of serum-free medium containing 0.1% of BSA were plated in a Transwell (BD Biosciences) that was placed in a 24 well plate. The bottom chamber contained 500 μl of complete DMEM medium. Cells were allowed to grow in the top chamber for 24 hr to facilitate cell migration from the top chamber to the bottom chamber. In the end of this incubation period, the membrane in the top chamber was fixed with 4% of paraformaldehyde for 30 min, washed with distill water, and air dried. Cells on the opposite side of the chamber (transmigrated) were stained with 0.1% of methyl violet (Dade-Behring, Newark, DE) for 30 min, washed, and viewed under an up-right microscope (x 400, NIKON). Cell migration was defined as numbers of cells on the membrane in 5 random review fields.

### Assay for cell invasion

Transwell chambers were coated with rat tail type I fibrillar collagen [15] for 30 min at 37°C. Excess fluid was removed and coated chambers were air dried for 1 hr in a sterile environment. Cells were suspended in 500 μl of serum-free DMEM medium containing 0.1% of BSA to a final density of 6×10^5^ and plated on the collagen matrix in a transwell that was placed in a 24 well plate. The bottom chamber contained 500 μl of complete DMEM medium. Cells remained on the membrane surface were gently removed 24 hr after cells were seeded and the membrane was fixed and stained for cells that had transmigrated through the collagen matrix to the opposite side of the transwell membrane.

### Statistical analyses

All data were analyzed using the SPSS 20.0 statistical program. Quantitative values were expressed as mean and SEM. The following statistical analyses were used in the study: Fisher’s exact test or chi-square test for defining the relationship between NME2 expression and pathological characteristics of cancer tissue; one way ANOVA for group comparison among parental cells and cells transfected with either a vehicle vector or a human *NME2* cDNA; and Pearson analysis for correlation between NME2 expression and differentiation and metastasis of cancer cells.

## Results

### Association of NME2 expression with histological characteristics of gastric cancer

**Fig 1:**
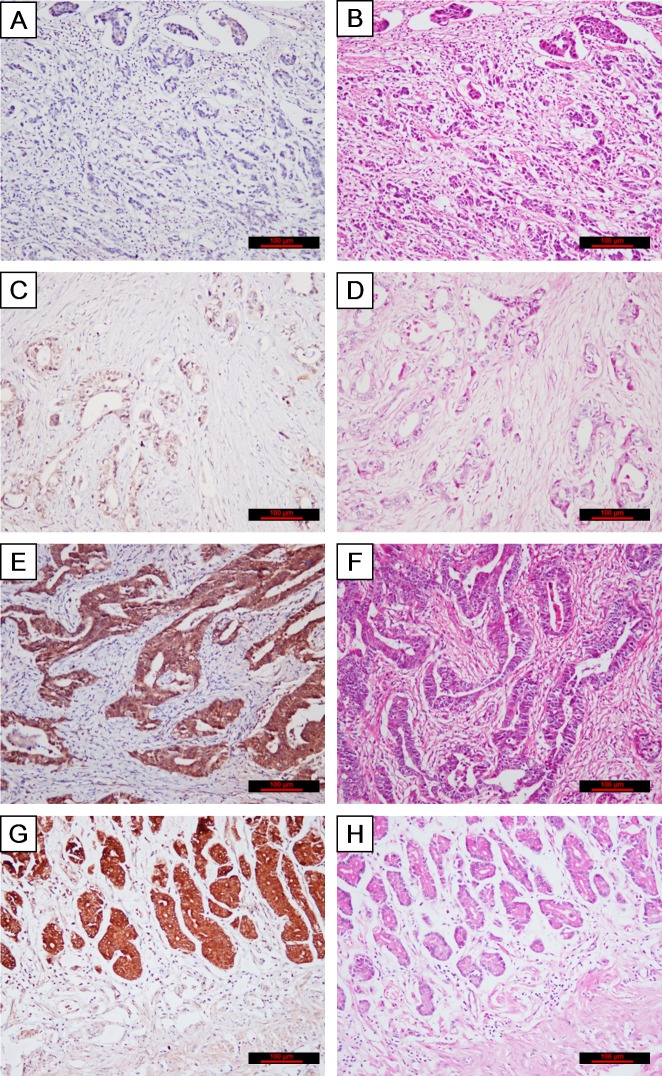
Expression of NME2 in gastric cancer. An immunohistochemical detection of NME2 expression in tissues from poorly (**A**), moderately (**C**), well (**E**) differentiated gastric cancer and adjacent normal tissue (**G**). The intensity of NME2 expression in well differentiated tissue is similar to that of normal tissue. The panel **B, D, F, H** are HE stain of tissues from the same sample blocks (Bar = 100 μm). These images are representative of tissue slides from 139 patients enrolled in this study.

We collected surgical specimen from 139 patients with gastric adenocarcinoma. Table 2 lists the demographic information of these patients and histological characteristics of cancer tissue from these patients. NME2 expression was weak or not detected in poorly differentiated tissue, but was intensive in well differentiated tissue at a level comparable to adjacent normal tissue (Fig. 1). A high level of NME2 expression was associated with a significantly reduced rate of metastasis to the lymph nodes (*p* = 0.038), but not with the size of primary tumor and depth of cancer invasion. A Pearson correlation analysis showed that NME2 expression was independently associated with rates of cell differentiation (r = 0.436, *p* = 0.000) and spread to the local lymph nodes (r = -0.281, *p* = 0.001, Table 1).

**Table 2:** Demographic and histological characterization of the patient cohort.

Pathological parameters	n	*NME2* positive (n)	Positivity (%)	*χ* ^2^	P
Gender				1.382	0.466
Male	111	99	89.2		
Female	28	27	96.3		
Age (yr)				0.243	0.757
≥55	94	86	91.5		
<55	45	40	88.9		
Differentiation				11.568	0.003**
Well differentiated	5	5	100.0		
Moderately differentiated	68	67	98.5		
Poorly differentiated and mucinous adenocarcinoma	66	54	81.8		
Primary tumor size(cm)				1.105	0.356
<4	93	86	92.5		
≥4	46	40	87.0		
Depth of invasion				3.240	0.356
T1	4	4	100.0		
T2	21	21	100.0		
T3	84	74	88.1		
T4	30	27	90.0		
Metastasis to lymph nodes				4.465	0.038*
No	33	33	100.0		
Yes	106	93	87.7		

Fisher’s exact test was used to calculate the association of *NME2* expression with gender, age, tumor diameter and lymph node metastasis. Comparisons between *NME2* expression and histologic characteristic were made using a chi-square test; *P<0.*05*, **P<0.*01*.

### Effect of NME2 on proliferation of gastric cancer cells in culture

**Fig 2:**
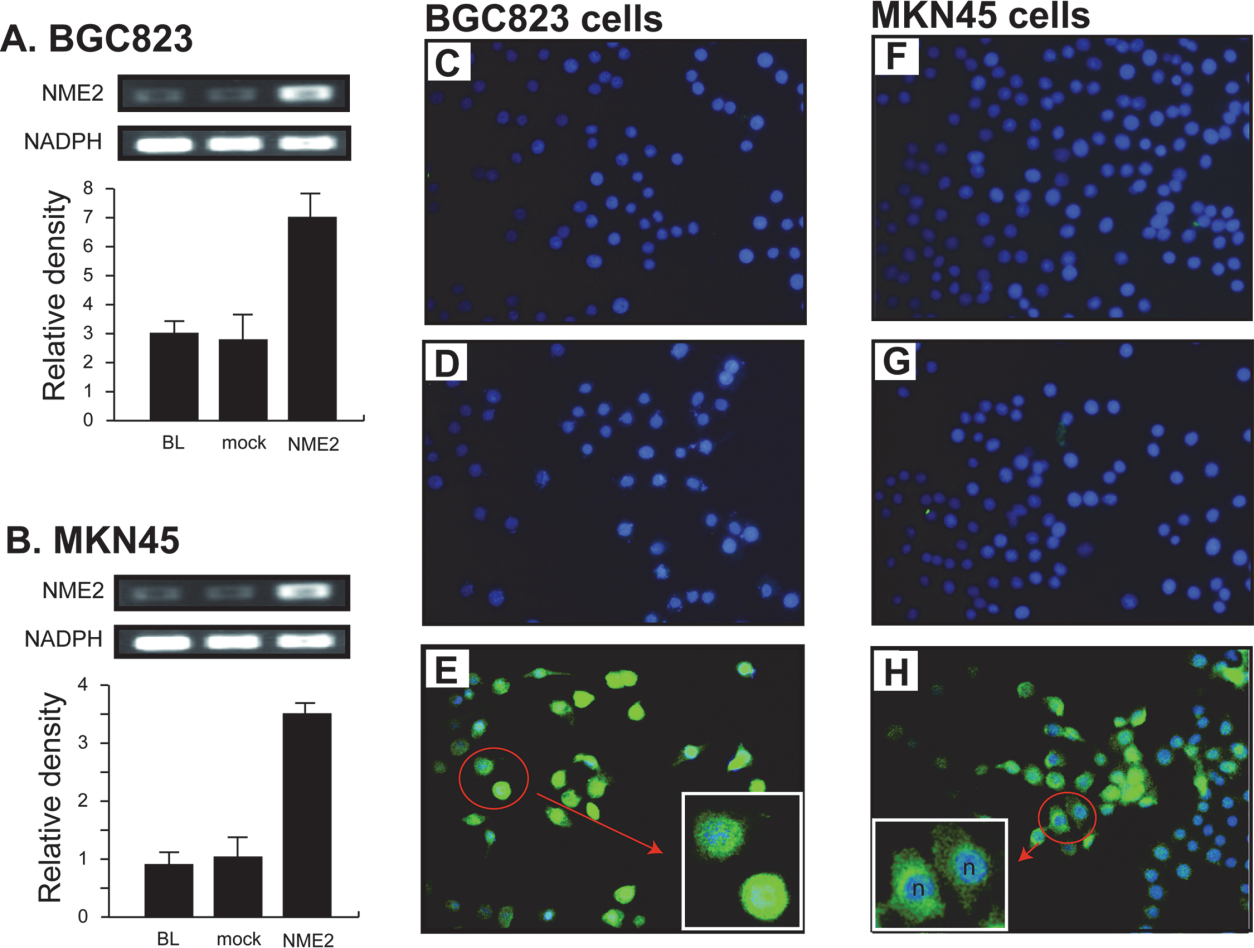
Stable expression of NME2 in two gastric cancer cell lines. **A & B:** RT-PCR profiles of *NME2* mRNA in mock and *NME2*-transfected BGC823 and MKN45 gastric cancer cells (parental BGC823 and MKN45 cells as baselines [BL]). Levels of the *NME2* mRNA from 3 separate sets of transfections were quantified by densitometry (ANOVA, n = 3, *p <0.05, compared to baseline). Immunofluorescence staining of NME2 in untransfected (**C & F**), mock (**D & G**) and NME2 (**E & H**) transfected BGC823 and MKN45 cells (Bar = 200 μm). A majority of NME2-transfected cells are stained positive for the kinase, which is intensively stained as dispersive and/or granular green fluorescence primarily in the cytoplasm, but also detectable in the nucleus. The patterns and levels of NME2 expression differ slightly in that NME2 in BGC823 cells was more evenly distributed in the cytoplasm with more intensive nucleus staining **(E insert),** whereas NME2 in MKN45 cells was more granular in distribution, with relatively less nucleus staining **(H insert, n: nucleus)**.

To further examine the association of NME2 expression with pathological characteristics of gastric cancer cells, we studied cells from the BGC823 and MKN45 gastric cancer lines, which expressed NME2 weakly at a level compared to poorly differentiated cancer tissue (Fig. 1). These cells were transfected with a human *NME2* cDNA to overexpress NME2 as quantitatively measured by an increase in the *NME2* mRNA (quantitative RT-PCR) and NME2 protein product (immunofluorescence, Fig. 2).

**Fig 3:**
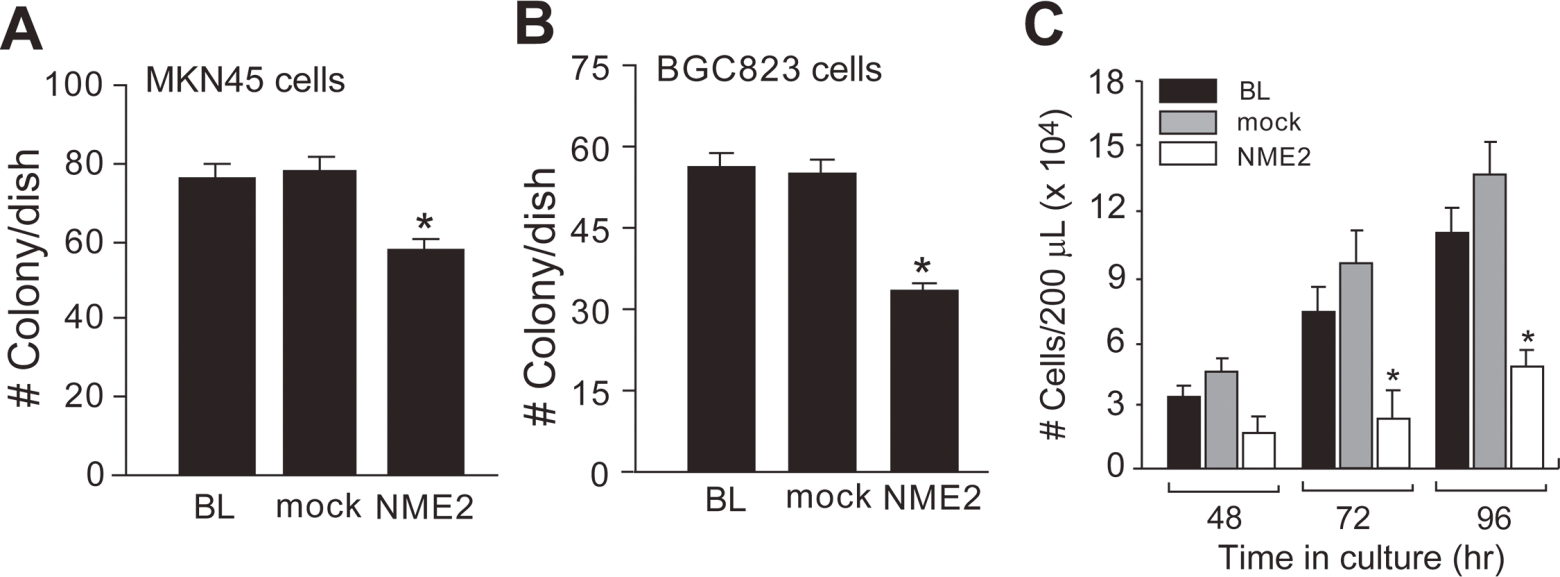
Effect of NME2 overexpression on cell proliferation. MKN45 **(A)** and BGC823 **(B)** cells transfected with a human *NME2* cDNA or a vehicle vector (mock), and untransfected cells (BL) were cultured for 14 days and then quantitatively analyzed for numbers of colonies formed (ANOVA, n = 3, *p <0.05 compared to parental cells [BL]). **(C)** Numbers of viable BCG823 cells in culture of these three types of cells for up to 96 hrs (ANOVA, n = 3/cell type at each time point, *p < 0.05 compared to parental cells).

We then investigated how overexpressing NME2 regulated cell growth by measuring the DNA ploidy and colony formation of these cells. We detected no difference in the DNA ploidy between cells overexpressing NME2 and those non-transfected and mock-transfected cells from both lines (Table 3 and S1 Fig.), suggesting that an increase in NME2 expression had no effect on cell cycles. However, NME2-overexpressing cells had a significantly reduced capacity to form colonies in culture as compared to sham transfected and non-transfected cells (Fig. 3). This observation was validated by results from a cell-counting method (Fig. 3C)

**Table 3:** Effect of *NME2* overexpression on cell cycle of gastric cancer cells*.

Cell	Stage of Cell Cycle			
	G0/G1	S	G2/M	S+G2
BGC823	66.0±2.0	27.9±1.6	6.4±0.3	34.3±1.5
BGC-mock	66.4±1.1	27.5±1.2	5.5±0.6	32.9±1.3
BGC-NME2	65.0±0.5	27.6±0.9	6.7±0.6	33.8±1.4
MKN45	58.2±0.9	36.1±0.4	6.3±0.3	42.4±0.8
MKN-mock	58,9±0.1	34.8±0.8	5.6±0.4	40.3±0.5
MKN-NME2	58.9±0.8	35.0±1.3	6.5±0.4	41.3±1.6

*ANOVA group analyses found no difference among untransfected cells and those transfected with either a vehicle vector or a human *NME2* cDNA.

### Effect of NME2 on cell migration and invasion

**Fig 4:**
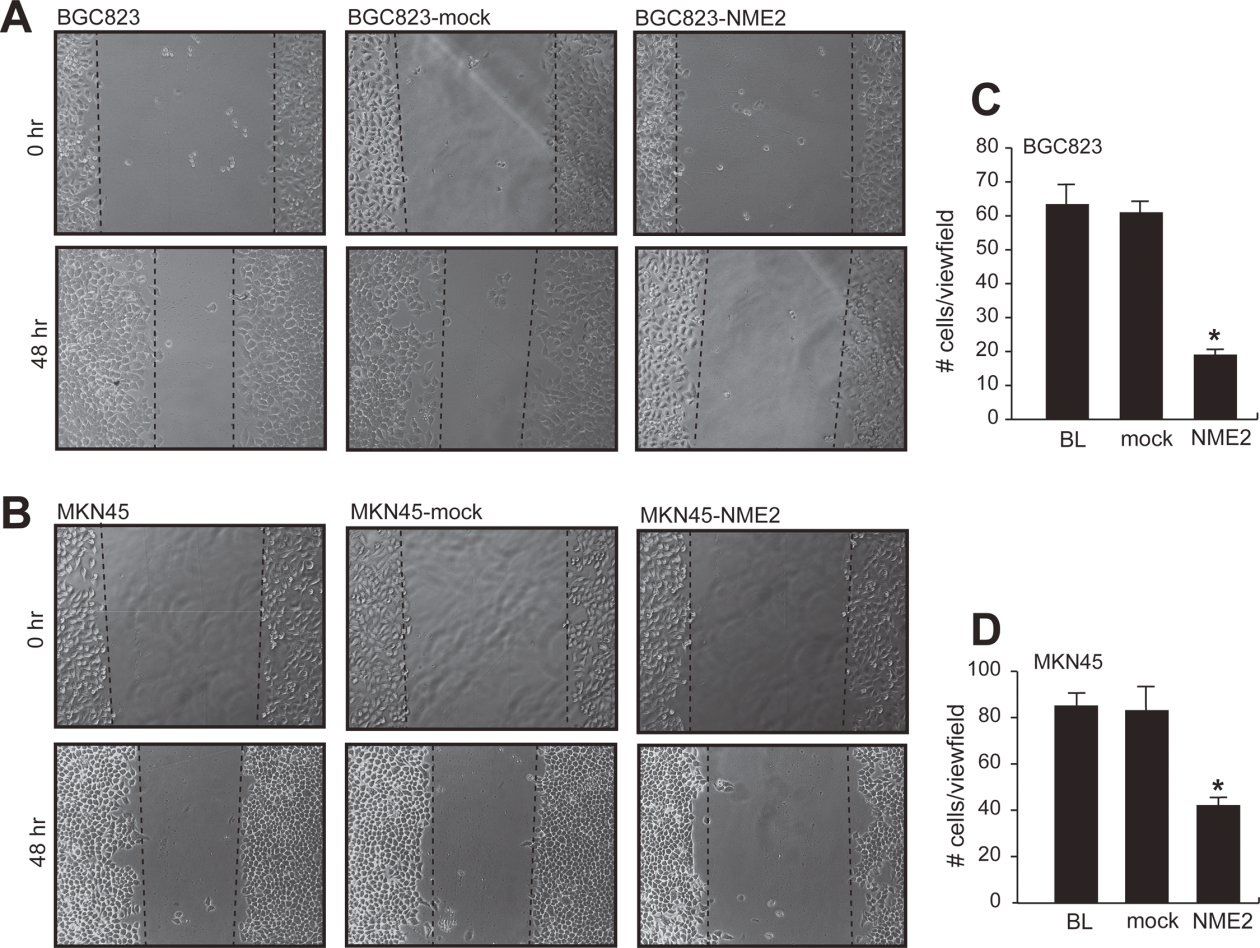
Effect of NME2 overexpression on cells migration. Parental BGC823 (**A**) and MKN45 (**B**) cells and cells that were stably transfected with either a vehicle vector (mock) or a human *NME2* cDNA migrate towards the scratch areas (defined by dot lines) 48hrs after injury (panels are representative images from 3 separate sets of experiments). **C & D**, The migration of these cells were also quantitatively measured in a transwell assay (ANOVA, n = 3/group, *p <0.05 compared to parental cells [BL]).

We next evaluated the ability of NME2-overexpressing cells to migrate because a directional migration is an important prerequisite for the invasion of cancer cells to surrounding tissue. This directional migration was examined using a wound-healing assay, which measures the rate of cells migration into the area of injury created by a sharp object. As shown in Fig. 4A and 4B, the width of an injured area after 48 hrs in cultures was greater for *NME2*-transfected cells than for non-transfected and mock-transfected cells. The slow migration was found in cells from both cell lines, suggesting that cells overexpressing NME2 migrated much slower. Because this wound healing assay is semi-quantitative, we also quantitatively measure the rate of cell migration using a Transwell assay. Consistent with the wound-healing assay, NME2 overexpressing cells migrated at ∼50% of non-transfected or mock-transfected cells (Fig. 4).

**Fig 5:**
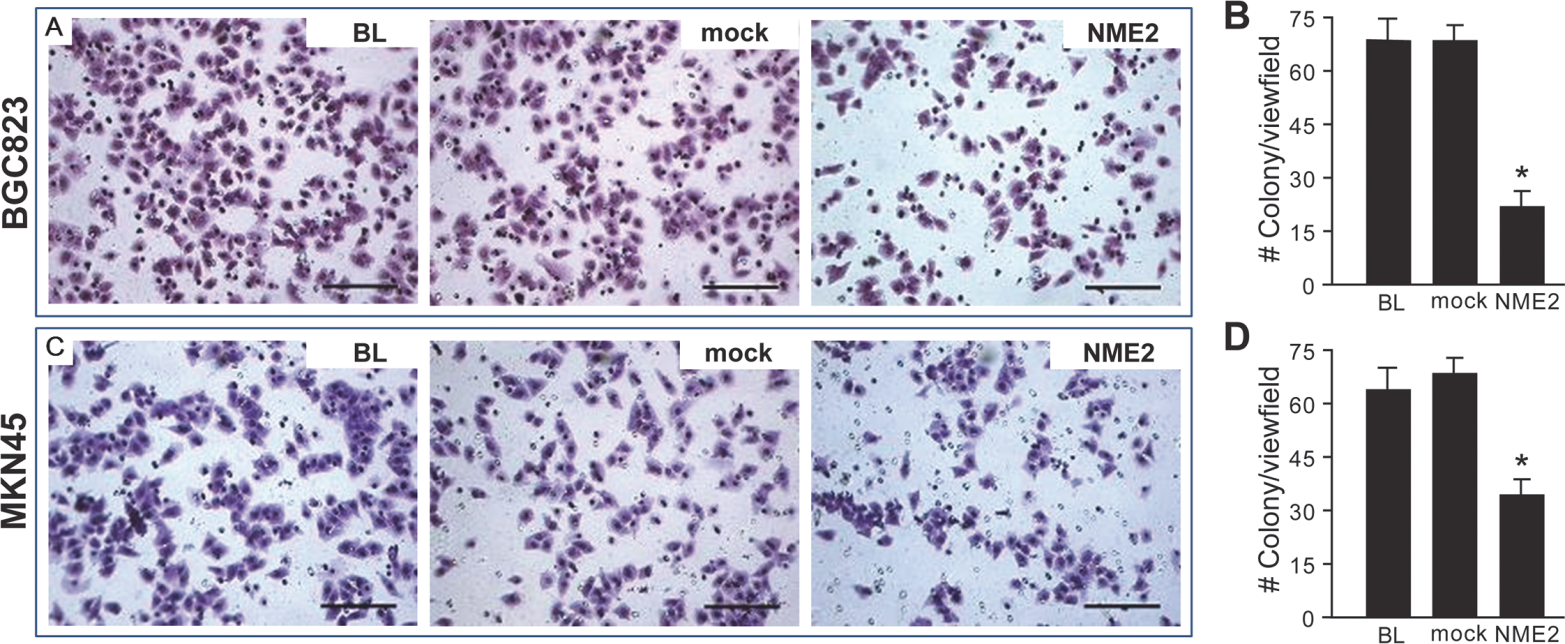
Effect of NME2 overexpression on cell invasion. Parental, mock- and *NME2* cDNA transfected BCG823 (**A**) and MKN45 (**B**) cells were measured for local invasion through the collagen matrix in a transwell assay. Panels **A** (BCM823) and **C** (MKN45) are snap images of cells that invaded through the collagen-matrix to the flip side of the membrane in transwell chambers. Panels **B** (BGC823) and **D** (MKN45) are quantitative summaries of multiple experiments (ANOVA, n = 3, *p <0.05 compared to parental cells [BL]).

A decrease in cell migration could potentially reduce the ability of these cells to invade surrounding tissue. To investigate this possibility, we measure the invasion of cancer cells to the collagen matrix in a well-established transwell chamber assay. BGC823 cells that overexpressed NME2 significantly reduced their ability to invade a collagen matrix to 60% of non-transfected and mock-transfected cells (Fig. 5). The rate of invasion was similarly reduced in MKN45 cells transfected with the *NME2* cDNA as compared to parental cells and those transfected with a vehicle vector (Fig. 5).

## Discussion

We have investigated the relationship between the NME2 expression and characteristics of gastric cancer in tissues surgically removed from patients and in cultured cells from two known gastric cancer lines. The study is necessary because *NME2* was initially identified as the first metastasis suppressor gene, but its ability to block cancer local invasion and remote metastasis appears to be cell type specific among cancers that have so far been studied [8–14]. For example, an analysis of 35 patients with thyroid papillary carcinoma and 11 of metastatic lymph nodes showed a significantly reduced level of the *NME1* mRNA in metastatic lymph nodes, whereas *NME2* mRNA was not changed in the original tumor and lymph nodes [14], suggesting differential roles of NME1 and NME2 in cancer invasion and metastasis. A meta-analysis of 278 patients with colon cancer, 177 with breast cancer, 137 with ovarian cancer and 77 with lung cancer also found a reduced level of NME2 expression in metastatic cancer as compared with non-metastatic cancer [17]. However, despite a negative association between NME1/NME2 expression and cancer invasion to the local lymph nodes and endometrial infiltration, NME expression was not associated with TNM scores and the differentiation of intrauterine membrane carcinoma and cervical cancer [16]. Here, we provide several lines of evidence that NME2 limits the proliferation, migration and invasion to the extracellular matrix of gastric cancer cells.

First, a higher level of NME2 expression is found in well-differentiated and less invasive tissue from gastric cancer surgically removed before chemotherapy and radiotherapy in a large patient cohort (Table 1 & Fig. 1). The metastasis of cancer to local lymph nodes was more frequently found in the cancer tissue that had a low level of NME2 expression and this correlation between NME2 expression and differentiation/metastasis in these patient tissues remains after the data were stratified for age, gender, the size of primary tumor and depth of invasion (Table 1 & 2). These findings are consistent with a recent report that NME2 binds the telomere repeat binding factor 2 in the nucleus to reduce telomerase activity [37], suggesting a mechanistic link between NME2 expression and survival of cancer cells.

Second, we examined effects of over-expressing NME2 on rates of proliferation, migration, and invasion to the extracellular matrix *in vitro* of cells from the two well-studied gastric cancer cell lines BGC823 and MKN45 [18–21]. The transfection with a human *NME2* cDNA resulted in a two-fold or greater level of NME2 expression in these cells as compared to mock transfected cells (Fig. 2). This NME2 overexpression did not alter DNA ploidy (Table 3), but significantly slowed the colony formation of these cells (Fig. 3), suggesting that overexpressing NME2 delays a transition from DNA replication to mitosis. This result is consistent with previous reports that NME2 does not affect the growth of cultured cancer cells and is not associated with the size of primary cancer implanted into animals [22–27]. However, it is inconsistent with reports that NME2 promotes [28,29] or reduces [30] the proliferation of other types of cancer cells. Exact reasons for the discrepancy remains to be further investigated, but these data suggest cell type-specific effects of the *NME2* gene on the pathogenesis of cancers.

Third, in contrast to a minimal effect on the proliferation of gastric cancer cells, the overexpression of NME2 results a significant reduction in cell migration and invasion through the collagen matrix as demonstrated in three distinct, but complementary assays (Figs. 4 & 5). Our data support previous findings of similar inhibitory effects on breast cancer [8], oral squamous cell cancer [28] and melanoma cells [9], although exceptions were again noted in hepatocellular [13,29] and colorectal carcinomas cells [31]. It remains to be determined as whether reduced migration and invasion of NME2-overexpressing cells are resulted from a decreased capability of these cells to proliferation.

Our results identified gastric cancer cells as sensitive to over-expressed NME2, but a critical question remains as what regulates these diverse NME2 activities among different types of cancer cells. One potential mechanism is that a NME2 activity depends on up- and downstream molecules that interact with NME2 in a given cell type. Multiple molecules have previously been identified to interact or regulate NME2, such as EGFR, *c-erbB-2*, *c-erbB-3* and sex steroid receptor in ovarian carcinomas [32]. Plakoglobin was reported to interact with NME2 to promote its expression in cells from human tongue squamous cell carcinoma [33]. NME2 was also found to interact with MDM2 (Mouse double minute 2 homolog) to reduce the motility of renal carcinoma cells [34]. MDM2 is an E3 ubiquitin-protein ligase and serves as a negative regulator of the p53 tumor suppressor. The relationship between *NME2* gene and genes of *myc* family appears to be more complicated. Products of the *NME1* and *NME2* genes have been suggested to be the downstream of the c-*myc* regulatory pathway [7] and involved in the down-regulation of cdc42 function in neuroblastoma [35]. NME2 is also reported to interact with the G-quadruplex DNA in the nuclease hypersensitive element of the *c-myc* promoter to induce c-myc expression [36]. A system biology approach to examine these specific pathways instead of individual molecules may be required to dissect roles of the *NME2* gene and its product in different types of cancers.

In summary, we have demonstrated that an overexpression of NME2 reduces the migration and invasion of gastric cancer cells to the cellular matrix *in vitro*. As a result, NME2 expression is associated with the well differentiated and less invasitve histology of gastric cancer. These results suggest that the *NME2* gene and its product may serve as a potential marker for predicting the invasiveness of gastric cancer and also as a therapeutic target that can be up-regulated through gene therapy.

## Supporting Information

S1 Fig Effect of NME2 overexpression on gastric cancer cells cell cycle. The DNA ploidy of cells transfected with *NME2* cDNA was analyzed by flow cytometry using a commercial kit according to the manufacturer’s instructions. The panels A-C were for parental BCG823 cells and those transfected with the *NME2* cDNA or vector. The panels D-F were for MKN45 cells with the same treatments.
(DOC)
