# Supplementary material for: NME2 Reduces Proliferation, Migration and Invasion of Gastric Cancer Cells to Limit Metastasis
Source: PLoS One. 2015 Feb 20;10(2):e0115968. doi: 10.1371/journal.pone.0115968 (PMC4336288; doi:10.1371/journal.pone.0115968)
Supplement: S1 Fig — The DNA ploidy of cells transfected with NME2 cDNA was analyzed by flow cytometry using a commercial kit according to the manufacturer’s instructions. The panels A-C were for parental BCG823 cells and those transfected with the NME2 cDNA or vector. The panels D-F were for MKN45 cells with the same treatments. (DOC) [file pone.0115968.s001.doc]

Supplemental Figure S1


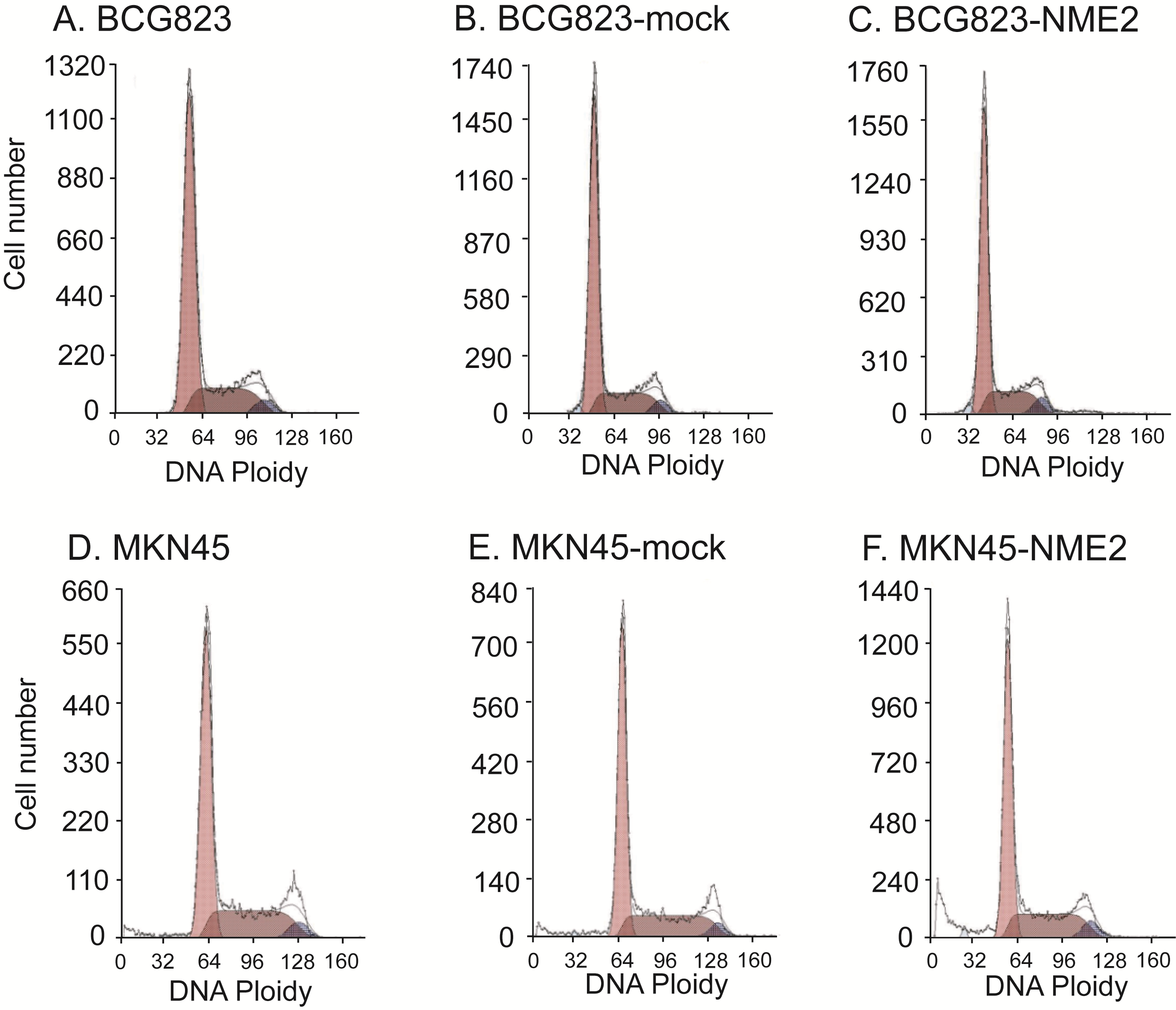


Figure S1: **Effect of NME2 overexpression on gastric cancer cells cell cycle: The** DNA ploidy of cells transfected with *NME2* cDNA was analyzed by flow cytometry using a commercial kit according to the manufacturer’s instructions. The panels A-C were for parental BCG823 cells and those transfected with the *NME2* cDNA or vector. The panels D-F were for MKN45 cells with the same treatments.
